# Streaming and redirection of lower acuity adult patients attending the ED: patient and staff experience

**DOI:** 10.1136/emermed-2024-214767

**Published:** 2025-06-26

**Authors:** Emily Louise Phillpotts, Jessica Coggins, Laura Goodwin, Sarah Voss, Edward Carlton, Rebecca Hoskins, Cathy Liddiard, Karen Butler, Laura Wilkinson, Grace Blows, Lisa Evans, Rebecca Macfarlane, Jonathan Benger

**Affiliations:** 1 University of the West of England School of Health and Social Wellbeing, Bristol, UK; 2 North Bristol NHS Trust, North Bristol NHS Trust, Bristol, UK; 3 Royal Surrey NHS Foundation Trust, Guildford, UK; 4 Epsom and Saint Helier University Hospitals NHS Trust, Carshalton, UK

**Keywords:** Emergency Medicine, emergency departments, triage

## Abstract

**Background:**

Streaming and redirection in the ED involves the assessment, triage and prioritisation of patients. Lower acuity patients can be streamed to alternative services within the hospital or redirected to off-site services with the aim of alleviating ED clinical pressures. This study aimed to understand staff, patient, family members and carers' experiences of streaming and redirection, including patients and staff who used an NHS web-based application.

**Methods:**

A semistructured interview study with staff working in the ED involved in streaming delivery and adult patients and family members who had attended the ED for conditions that could be safely managed in lower acuity settings. Participants were recruited from two NHS Hospital sites in England, one using a web-based NHS urgent care self-service product (digital tool) and one using a nurse-led streaming model. Recruitment took place between August and December 2023.

**Results:**

28 interviews were completed. Participants across both sites commented on the challenges of streaming and redirection and confusion around where patients needed to go for what conditions. Patients and staff felt that the lack of capacity in alternative services limited the effectiveness of streaming and redirection. Three themes developed: ‘it’s too muddy’: disruption to the flow of care, balancing privacy and efficiency in streaming and redirection, pressures in the wider healthcare system beyond the ED.

**Conclusions:**

This research has implications for understanding patient and staff experiences of streaming and redirection approaches, and the need for clear communication and navigation when utilising digital technologies in the ED.

**WHAT IS ALREADY KNOWN ON THIS TOPIC**

- The aim of streaming and redirection is to increase capacity in the ED, enhance patient flow and improve access to health services by redirecting low acuity patients to other sources of care.
- An NHS web-based urgent care self-service digital tool has begun to be introduced to EDs in England to allow patients to determine the best site for care.
- The experiences of patients and staff with streaming and redirection have not been explored.

**WHAT THIS STUDY ADDS**

- In this qualitative study, patients described confusion about where to go, who they should be seeing, whether or not the digital tool was in use.
- Additional confusion arose as the digital tool still required oversight from a streamer nurse.
- Staff emphasised the importance of clinical intuition in making streaming and redirection decisions.
- Patients and staff noted that streaming and redirection efforts are limited due to insufficient capacity within the wider healthcare system.

**HOW THIS STUDY MIGHT AFFECT RESEARCH, PRACTICE OR POLICY**

- This research describes the need for clearer communication and navigation processes for patients and staff when making streaming and redirection decisions.
- The study has implications for the development of web-based applications and their integration with existing protocols and procedures to support assessment, evaluation, triage and streaming within the ED.

## Introduction

 The Royal College of Emergency Medicine defines streaming as ‘the process of allocating patients to different physical areas, services, pathways or processes to improve efficiency and effectiveness’, while redirection is defined as ‘the process of referring patients who do not require emergency care away from the ED‘.^1^ Streaming involves directing patients to specialist internal services while redirection involves redirecting patients to alternative services or self-care off-site.^1 2^

While patients can access an ED directly, or arrive by ambulance, they can also use the NHS 111 service, a telephone and digital triage system, that directs patients to the most appropriate service for their needs.^3^ Recently, some hospital trusts in the UK have implemented a digital tool in the ED that uses the NHS111 algorithms. This requires patients to answer a series of questions about their symptoms to support prioritisation of care internally or redirection to an alternative service.^4^

The use of digital applications for streaming and redirection has the potential to alleviate pressures on both clinical and non-clinical ED staff and reduce ED waiting times.^5^,^7^ Nevertheless, the effectiveness of different models of streaming depends on factors such as demand, physical space and hospital resources.^8^ It is, therefore, important to explore both patient and staff experiences of streaming and redirection within the ED to guide future decisions about how these services are designed and delivered and inform future commissioning decisions and service delivery.

The evidence surrounding the experience of streaming and redirection from a patient or staff perspective is limited. Patients are not always suitable for redirection, many patients refuse redirection, and there are additional costs to streaming including staffing and training requirements.^9^ Feasibility studies have revealed that having a general practitioner streaming improved waiting times and reduced the number of patients with minor injuries or illness waiting more than 4 hours for admission.^10^ This research found that 86% of patients seen by a general practitioner in the ED for streaming and redirection needed no further intervention and were discharged.^10^ Fast discharge was a significant determinant of patient satisfaction.^10^ Nevertheless, a systematic review of interventions to improve patient flow in the ED found that streaming and redirection did not improve the flow of care for patients.^11^

There are few studies evaluating technological approaches to streaming and redirection which could reduce the need for an individual to perform streaming and redirection. A cohort observational study investigated the safety of redirection for low acuity patients using an electronic clinical support system. The streamer nurse worked in tandem with the electronic clinical decision support system to evaluate if the patient was suitable for redirection. The study revealed that patients were generally satisfied to be redirected, noting that all were redirected to a site within 5 km of the original ED attended.^12^ However, while the study looked at the impact of being redirected on patient satisfaction and return rates to the ED, it did not capture patient or staff experiences of using a digital application for streaming and redirection.

This study aimed to understand staff, patients, family and carers’ experiences of streaming and redirection. This study recruited participants from two sites, one using an existing model of streaming and redirection in the southwest of England and one site using a digital tool in the southeast of England.

## Methods

### Setting

This study was based in the EDs of two NHS Hospital Trusts in the southwest and the southeast of England, selected purposively for their two different methods of streaming and redirection (traditional vs digital). The southwest site used a traditional approach with a trained ED nurse who was stationed at reception with the specific role of rapid assessment and potential redirection of patients. All patients attending the ED were first registered by an ED receptionist. Patients had a brief discussion with a senior nurse at reception and, if appropriate, were asked to see a dedicated ‘redirection’ nurse. Those identified by a brief initial discussion with a senior ED nurse at reception for potential redirection were sent to a dedicated ‘redirection’ nurse. The redirection nurse then completed an assessment based on the Manchester Triage system, including recording a set of physiological observations. Following assessment, patients were either redirected, streamed or retained in the ED for further evaluation and treatment as appropriate.

The southeast site had recently implemented the NHS urgent care self-service tool; a web-based application (digital tool) used within the ED which most patients were encouraged to use as appropriate (with the exception of patients who had already spoken to NHS111 or patients experiencing symptoms of stroke, chest pain or heavy bleeding). The urgent care self-service product digital tool was presented to patients on one of four iPads within the ED foyer and asked the same questions as are used in NHS111. A streamer nurse stood near the iPads to support patients in completing the questions. Once completed, a destination was provided (eg, minor injuries unit). The streamer nurse then reviewed the patient information and made a digital note indicating if they agreed or disagreed with the recommendation provided by the digital tool. Patients then registered with the most appropriate service, as recommended or confirmed by the streamer nurse. At the time of this study, the iPads were an extra step for patients and staff and were being trialled, with the information provided by the streamer nurse used to train the system to make more accurate recommendations.

The two departments were located in different regions of England with different numbers of patient attendance, different case mixes and service and staff configuration. One site is a Major Trauma Centre and one site is a Trauma Unit with patient volumes of >100 000 and <20 000 in 2023–2024, respectively.

### Patient and public involvement

A dedicated patient and public involvement (PPI) group of six people comprised people who had experience of attending the ED was established to support the design of the study. The group provided feedback on the study aims, design, participant documentation, interview topic guide and a review of the initial findings.

### Study design

The study adopted a descriptive qualitative approach^13^ using semistructured interviews to examine inductively the subjective accounts of staff, patients and family members and carers in relation to their experiences of streaming and redirection for patients attending the ED for minor injury/illness. In the context of this study, minor injury/illness was defined as conditions that could be safely managed in lower acuity settings (eg, general practice, minor injury unit, pharmacy).

### Recruitment and consent

Recruitment took place from August to December 2023. The study aimed to recruit up to 30 patients or family members, and up to 20 ED staff at each site, guided by information power with a narrow aim and specific study population. ^14^

Staff over the age of 18 years, working in the two EDs and all adult patients and family members/carers attending those EDs for minor illness or injury were eligible to participate. Eligible staff participants were sent an email by a local collaborator at each hospital site and invited to contact the study team.

Patients were recruited through convenience sampling by the local study team during their visit to the ED. Eligible patients were provided with a participant information sheet (PIS) and completed a consent to contact form either on paper or through a QR code and provided their contact details. The PIS provided information about the research aims and rationale for the study. If the patient lacked capacity and was unable to consent to participate, the attending family member or carer was invited to participate and provided with the PIS and consent form. Consenting patients or caregivers were contacted by the research team to arrange a time and date to conduct an interview within 7–10 days of ED attendance.

Potential interview participants also received a PIS, privacy notice and consent form from the research team via email. The electronic consent form was completed prior to the interview, and verbal confirmation of consent was also audio recorded at the start of the interview.

### Data collection

The PIS, consent form and topic guides were developed and piloted in collaboration with the study team (including clinicians local to each site) and the PPI group. The topic guide for staff focused on their experiences of facilitating streaming and redirection. The topic guide for patients focused on their experience of attending the ED and being streamed or redirected. Questions for family members focused on their experience of streaming and redirection and their experience of supporting someone to use the digital tool.

Interviews were conducted between October and December 2023 by an experienced female qualitative research associate (JC; qualitatively trained at masters level, non-clinical researcher with several years of experience in health research). Interviews were conducted after ED attendance either by phone or video call (on Microsoft Teams) according to participant preference. Interviews were audio or audiovisually recorded using the record function on Microsoft Teams. Audio files were transcribed verbatim by a university-approved transcriber.

### Data analysis

Anonymised interview transcripts were coded and analysed by ELP, a female research associate, supported by NVivo V.14 Qualitative Software. Data were analysed thematically through an iterative process of data reduction, constant comparison, organisation and understanding.^15^ The researchers read the transcripts several times and then coded sections of text to represent instances of a concept. A thematic map was used to combine codes and create more developed themes. The proposed themes were cross-checked with members of the study team who conducted the interviews to check the interpretation of the data.

### Reflexivity statement

Both the interviewer (JC) and researcher analysing the data (ELP) are trained in qualitative research methods. Our philosophical stance is broadly constructivist, acknowledging that knowledge is co-constructed through the interactions between the researcher and the participant. We acknowledge that our backgrounds in health research and familiarity with emergency care research may have influenced the framing of the interview questions, interpretations of the data and ultimately, the final themes. Throughout the development of the topic guide and through data collection, members of the wider study team were consulted, given their clinical expertise, to ensure the assumptions and interpretation of the data were sound.

## Results

### Participant characteristics

In total, 73 participants expressed an interest; however, due to difficulties in consolidating recruitment, 28 participants in total completed qualitative interviews. This included 18 members of staff, nine patients and one family member with 14 participants from each site, who had been seen by a redirection nurse and/or used the digital tool to be streamed (table 1). Participant characteristics were grouped together and comprised nurses, doctors, patients, family members and ‘other’ (table 2). Interviews lasted an average of 18 min.

**Table 1:** Number of staff, patients and family members/carers from each site

	Staff	Patients	Family members/carers	Total
Southwest	10	3	1	**14**
Southeast	8	6	0	**14**
	**18**	**9**	**1**	**28**

Participant group type and total number of participants recruited to each group in bold.

**Table 2:** Characteristics of participants in the study (P indicates participant number)

	Southwest	Southeast
Nurse	P13	P1, P9
Senior nurse	P3, P8, P17	P5, P7, P20, P22
Doctor	P14, P16, P18, P23	P2
**Other (staff)**	P15, P19,	P4
Patient	P12, P24, P25,	P6, P10, P11, P21, P26, P28
Family member	P27	

**Figure 1:**
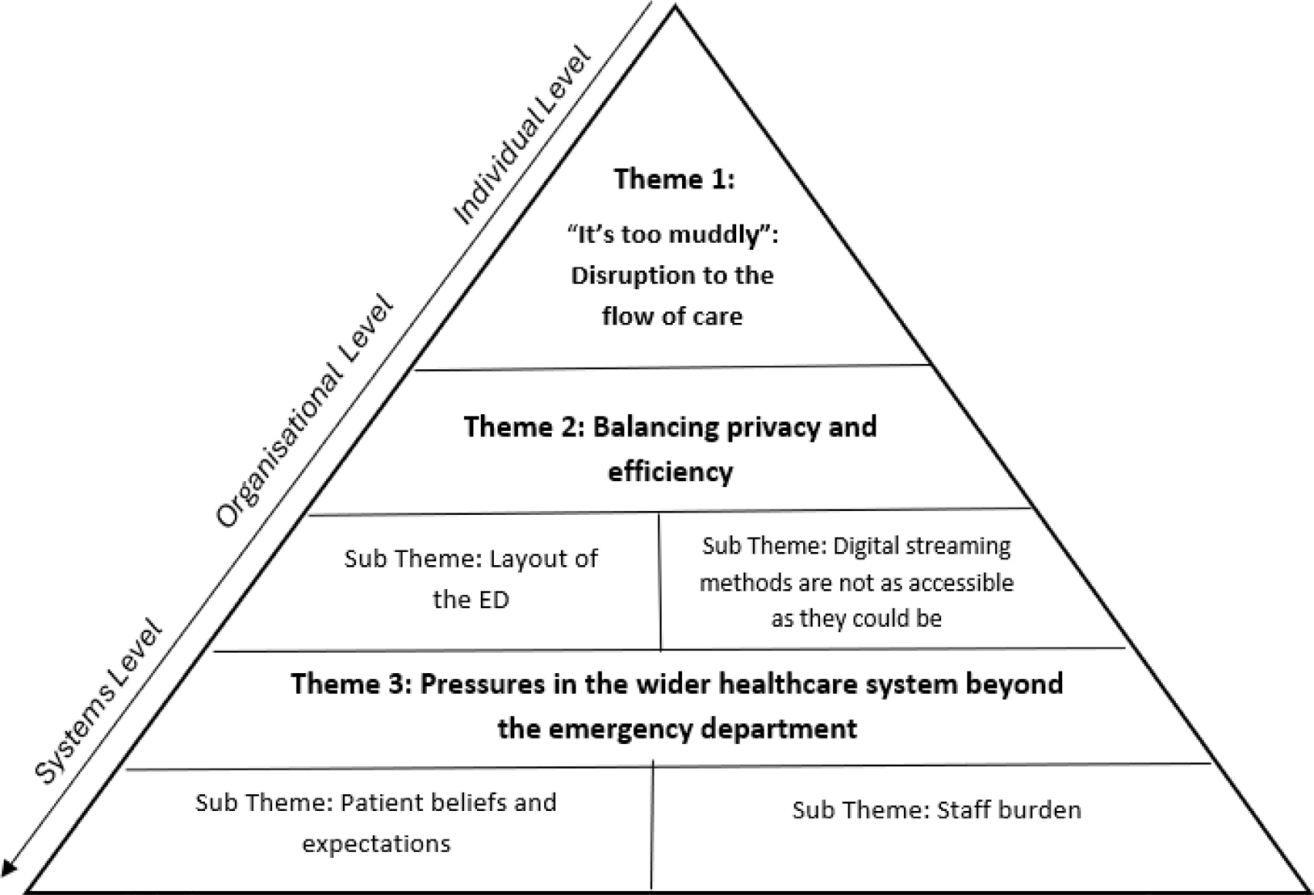
Diagram showing key themes and subthemes.

Three themes were developed with four subthemes to describe patient and staff experiences of streaming and redirection across two EDs in the UK. Patients and staff described their experiences at individual level, an organisation level and at a systems level (figure 1).

### Theme 1: ‘It’s too muddy’: Disruption to the flow of care

Patients commented on their lack of knowledge about which services to access for minor injuries/illness. Patients acknowledged that they had limited awareness of what services were appropriate for which conditions, and where, when and how to access these services. Patients were unclear on the difference between the ED and the minor injury unit. In the Southeast site, these services were located very close to each other. As such, participants were not necessarily aware they were being ‘streamed’ or ‘redirected’.

“I know that in theory there are various clinics for different things. But I couldn't tell you what they are or how to access them” (Patient 6, Southeast).

“I don’t know if there is another place that is more minor injuries or urgent care. I think there is like an UCC (urgent care centre) actually in some hospitals. So, I actually don’t know really. I am not sure where to find out either.” (Family Member, 27, Southwest)

Staff indicated that there is an element of clinical intuition; staff use their tacit knowledge and experience and can physically see the condition of the patient, which is important to ensure that patients are sent to the right places. As such, in its current format, the digital tool still requires oversight from the streamer nurse, which was seen by some as a duplication of effort.

“If someone says they’re dizzy for example, if they click yes, I’m dizzy on the iPad, then it will say, are you able to walk four steps? They click yes, and then it will say, ok go to urgent care, but really a dizzy person, regardless of age…their pulse rate could be over two hundred. So…they need to be in majors or even resus, whereas the iPad doesn’t ask for any observations to be taken, and it has the potential to be quite dangerous, I think” (P22, Senior Nurse, Southeast)

Additionally, confusion sometimes arose when patients were given information from the digital tool, which had not then been confirmed with the streamer nurse. This could result in patients being streamed to the wrong place and then sent back to ED again. This led to frustration, where patients were confused about where they needed to go, what the purpose of filling out the questions on the tool was, and who they could ask for assistance.

So, that was a bit annoying, that I walked, or hobbled to another department to then be sent back. And then of course, once I did check in, I then went into another area to sit, and as soon as I’d sat down, someone said, oh no you need to go, and then they sent me back to the department that I’d originally gone to. (Patient 26, Southeast)

### Theme 2: Balancing privacy and efficiency in streaming and redirection

The purpose of streaming and redirection is to increase capacity in the ED. However, participants in this study noted that there can be barriers to patients engaging with streaming and redirection efforts regardless of approach.

### Subtheme:The layout of the ED has an influence on clinical decision-making

Staff indicated that, even with the support of the digital tool, streaming and redirection remain resource intensive and require the streamer nurse to support patients while also checking the digital tool was providing the correct information. Staff at both sites, regardless of streaming approach, expressed concerns about privacy in the ED and the extent to which patients would disclose symptoms. This could have consequences for where the patient is streamed or redirected to.

If they come in with private, personal issues, or something like miscarriage or mental health issues, it’s just not nice for them to be talking about it in front of other people (P22, Senior Nurse, Southeast)

The information you get is only as accurate as the a) questioning and b) the willingness of the patient to speak out in front of, you know, what can be a really busy waiting (room). There’s lots of people near you. And you know, certainly from sort of any mental health stuff or domestic violence things or whatever, I, I would you know, I’d be amazed if, if patients were happy to disclose that kind of stuff over a counter essentially. (P15, Staff (Other), Southwest)

### Subtheme: Digital streaming methods are not as accessible as they could be

Staff reported that the digital tool is not accessible to all patients, for example, older patients, patients with learning difficulties, disabilities and people who are visually impaired. Staff also described technical issues when using the digital tool that meant that oftentimes, staff and patients would stop trying to use it altogether. The use of the digital tool was seen by some staff as compounding and lengthening the already stressful experience of attending the ED for some patients.

I think it’s, the questions are too long for patients, especially the ones that are genuinely unwell, needing to see a doctor, they spend about two to four minutes, filling out details on an iPad, telling the iPad what’s wrong with them and then they have to go to the streamer nurse, give the streamer nurse the same information, and then go to reception, give all their details to reception, just to get booked-in (P20, Senior Nurse, Southeast)

Staff commented that the digital tool is time-consuming, and patients question the length and relevance of the questions asked. Staff commented on how the format of the questions may not be accessible with little functionality to change the settings to suit the needs of patients.

Elderly people aren’t great with touch screen, the writing is quite small and you can’t make it any bigger, and also people with limited use of English, they don’t work for (P22 Senior Nurse, Southeast)

### Theme 3: Pressures in the wider healthcare system beyond the ED

Participants emphasised the wider issues within the healthcare system that affect engagement with streaming and redirection in the ED and the reasons why patients attend the ED. Participants described patient expectations of the ED and the implications for staff burden.

### Subtheme: Patient beliefs and expectations of treatment

Patient accounts of their ED attendance focused on their experiences of care and triage, rather than experiences relating to whether they saw a nurse or used the digital tool. When asked explicitly about the use of the digital tool, patients suggested that if individuals had travelled to attend the ED, they had attended because they believed it was the most appropriate place for them and as such may have an expectation to be seen by a healthcare professional there.

Everyone’s got an opinion of how best to care for themselves, I guess, and so, I could imagine some people being very frustrated because they’re hoping they they’re going to come and […] get seen to (Patient 12, Southwest)

This could have implications for how patients choose to engage in health services in the future. Staff described how patients struggle to access alternative services in the first instance and therefore present to the ED. Staff described how patients may get stuck in a cycle or disengage with health services. Staff felt that the pressures on the system limited the effectiveness of streaming and redirection efforts.

For those who feel they’ve been redirected inappropriately, it may put them off going there. Oh they are just going to send us away back to the pharmacy, I might as well not even go, I might as well just go to the pharmacy myself. Then they go to the pharmacist who says no, I think you should be seen by your GP or your doctor and they can’t get a GP appointment, then they end up having to come to A&E and then they are redirected. You know, it is just a cycle, so some individuals will just take a step back and think I am just not going to try. (P4, Staff (Other), Southeast)

### Subtheme: Staff burden

Staff from both sites, regardless of approach, expressed that the potential benefits of streaming and redirection were not as effective at alleviating capacity within the ED as they could be. This could have been due to pressures on the whole healthcare system or specific issues with the implementation of the digital tool.

Once they’re here, you spend as much time trying to sort them out and redirecting them as you might do just seeing them… (P15, Staff (Other), Southwest)

The iPad doesn’t help me at all, in fact it makes my life more difficult (P1, Nurse, Southeast)

Staff across both sites, regardless of streaming and redirection method, described how streaming and redirection is not a popular role within the team and the challenges inherent in telling patients that they need to go elsewhere. As such, staff described an increase in verbal aggression, which staff implied was attributable to increasing difficulty in accessing health services.

It’s not a very popular role, […] all you’re doing is asking patients to leave and not really giving them a viable alternative (P15, Staff (Other) Southwest)

## Discussion

This qualitative interview study investigated the experiences of 28 patients, staff and family members attending the ED for minor injury/illness. Participants were recruited from two NHS Hospital Trusts in England who implemented different methods of streaming and redirection. Participants shared their experiences across three levels. At an individual level, participants commented that streaming and redirection processes can be confusing for patients. Additionally, staff commented on the importance of clinical intuition in making streaming and redirection decisions, and that this is not replicated in the digital tool. At an organisational level, across both sites, participants felt the layout of the ED and the lack of designated space for the streamer nurse to have private conversations with patients was problematic. Additionally, participants noted that the digital tool is not necessarily as accessible as it could be, and the length and relevance of questions asked was frustrating for patients and challenging for staff. Finally, staff and patients, regardless of approach, noted that current streaming and redirection methods are limited by pressures at a systems level.

Patients described how they were unsure about where they needed to go for their illness/injury, where they were being streamed to and who they could ask for help. In the Southeast site, the digital tool was still in a trial implementation phase and as such, patients would complete the questions on the digital tool and then also be assessed by the streamer nurse. This was an important part of ‘training’ the digital tool.

Staff felt that clinical intuition was an important part of the streaming and redirection process that could not yet be replicated.^16^ Thus, the presence of a streamer nurse to support digital approaches to streaming and redirection was described as a clinical ‘safety net’,^17^ to ensure patients were not incorrectly redirected. Previous research has found that it is important to consider intuition and experience when making streaming and redirection decisions.^17^ Therefore, the allocation of staff needs to consider the balance of clinical confidence and competence with the effective use of the time of experienced nurses.^16^

There were also important concerns that the digital approach to streaming isn’t necessarily feasible in the ED. The digital tool uses the same questions and format as the NHS111 system. Previous research on patient engagement with NHS111 indicates that while patients are generally satisfied with the 111 service, they do not always understand the relevance and extent of the questions they are asked.^18 19^ This is reflected in the findings from the current study where patients questioned the length and relevance of the questions they were required to answer, especially those patients who both completed the digital tool and then were seen by a streamer nurse.

Streaming and redirection can be efficient and reduce waiting times for patients.^20^ However, staff and patients across both sites, regardless of streaming method, commented on the challenges of implementing this into routine practice. Staff in this study implied that the layout of the ED and the use of the digital tool could discourage the disclosure of information by patients. This is reflected in previous work which has found that a lack of privacy in the ED has the potential to limit the ability of staff to build rapport with patients.^21^ Previous research suggests that individuals do not volunteer their motivation for seeking care unless the clinician specifically asks for it.^22^ Staff in this study felt that the lack of dedicated space for streaming and redirection meant that the opportunity to ask about this is missed. The lack of designated space for streaming, alongside the lack of physical and human resources, negatively impacts patient experiences of care in this context.^23^

Additionally, patients suggested that there is limited functionality associated with using the digital tool, increasing the number of patients who need to be assessed by the streamer nurse instead. This study showed that this is frustrating for both staff and patients and could also have an impact on the timeliness and flow of care for minor injury and illness.

Finally, patients in this study implied that people may come to the ED with expectations and beliefs about their care and that if they have travelled to the ED, they expect to be seen there, rather than redirected elsewhere. Previous research has suggested that where these expectations are not met, this can have a negative consequence for treatment adherence and future engagement with health services.^24^ While healthcare professionals will be familiar with internal hospital departments and the ED, these will be unfamiliar to patients and as such, communication between patients and healthcare professionals is important to mitigate frustrations and manage expectations.^22^ Staff in this study explained how, regardless of streaming method, streaming and redirection was not a popular role within the team. Streaming and redirection roles divert nurses away from other ED work and place a significant psychological and physical burden on nurses.^25 26^

Important limitations of this study include the fact that we were unable to recruit any patients who had been redirected away from the ED, and thus the results do not reflect the experiences of this group. The initial recruitment targets were not met, and so the sample is a relatively small convenience sample of those willing to participate, recruited in two areas of England. It should, therefore, not be considered representative of patients, carers and staff in all areas of England or all ED services. The two participating EDs have different characteristics and cannot be compared directly. Further research may choose to adopt a longitudinal mixed-methods approach to investigate patient journeys through streaming and redirection pathways and identify outcome measures that can provide a comprehensive assessment of patient outcomes, safety and experience. Future research should also aim to recruit patients who have experience of redirection from the ED to enhance the findings presented here and offer an additional, important perspective.

## Conclusions

This study assessed patient and staff experiences of streaming and redirection within the ED. At the time of our research, the digital tool under study was subject to oversight from a streamer nurse, which can lead to frustration for staff and confusion for patients. Patients and staff indicated that further development of web-based interventions to be more accessible to patients is required. Patients commented on their experiences of accessing alternative healthcare services, and some feel negatively about being redirected away from the ED with the risk of experiencing further diversion. Regardless of streaming approach, participants felt that streaming and redirection efforts are limited in their effectiveness due to ongoing pressures within the wider healthcare system.

## Data Availability

Data are available on reasonable request.
